# Factors influencing healthcare personnel decision making to work with respiratory symptoms during the COVID-19 pandemic

**DOI:** 10.1017/ash.2023.301

**Published:** 2023-09-29

**Authors:** Rachel Meyer, Michael Kessler, Daniel Shirley, Linda Stevens, Fauzia Osman, Nasia Safdar

## Abstract

**Background:** Amid the COVID-19 pandemic, healthcare systems were stretched thin, with staffing shortages posing substantial challenges. Limiting spread of COVID-19 among healthcare professionals (HCP) is paramount to preventing exacerbation of such shortages, but strategies are highly dependent on HCP self-screening for symptoms and isolating when present. We examined HCP perceptions of barriers and factors that facilitate staying home when experiencing respiratory symptoms. **Methods:** At an academic tertiary-care referral center, in inpatient and ambulatory settings, we conducted an anonymous electronic survey between March 11, 2022, and April 12, 2022. Using logistic regression analysis, we analyzed predictors of employees reporting to work with respiratory symptoms using STATA and SAS software. **Results:** In total, 1,185 individuals including 829 clinical staff and 356 nonclinical staff responded to the survey. When excluding participants who reported working “remotely” (N = 381) and those who reported being unsure of whether they had worked with symptoms (N = 14), the prevalence of working with respiratory symptoms was 63%. There was no significant difference between clinical and nonclinical staff (OR, 1.1; 95% CI, 0.8–1.5; *P* = .60). Increasing number of years of service was protective against working with symptoms, achieving statistically significance in multivariable analysis after 16 years. Compared to those having worked <1 year, the odds ratios of working with symptoms were 0.32 (95% CI, 0.16–0.65; *P* = .002), 0.33 (95% CI, 0.15–0.74; *P* = .007), and 0.32 (95% CI, 0.13–0.79; *P* = .007) for those working 16–20 years, 21–25 years, and ≥26 years, respectively. More than half of HCP who worked with symptoms identified being understaffed (56.9%), having mild symptoms (55.3%), and sense of responsibility (55.1%) as reasons to work with respiratory symptoms. The following barriers, or reasons to work with symptoms, were more commonly identified as significant by those who worked with symptoms compared to those who did not: being understaffed (OR, 1.87; 95% CI, 1.35–2.58; *P* ≤ .001), having mild symptoms (OR, 1.96; 95% CI, 1.42–2.71; *P* < .001), and lack of support from management (OR, 1.84; 95% CI, 1.07–3.18; *P* = .03). **Conclusions:** Working with respiratory symptoms is prevalent in clinical and nonclinical HCP. Those with fewer years of work experience appear to be more susceptible to misconceptions and pressures to work despite respiratory symptoms. Messaging should stress support from leadership and the significance of even mild respiratory symptoms and should emphasize responsibility to patients and colleagues to stay home with respiratory symptoms. Strategies to ensure adequate staffing and sick leave may also be high yield.

**Figure:**
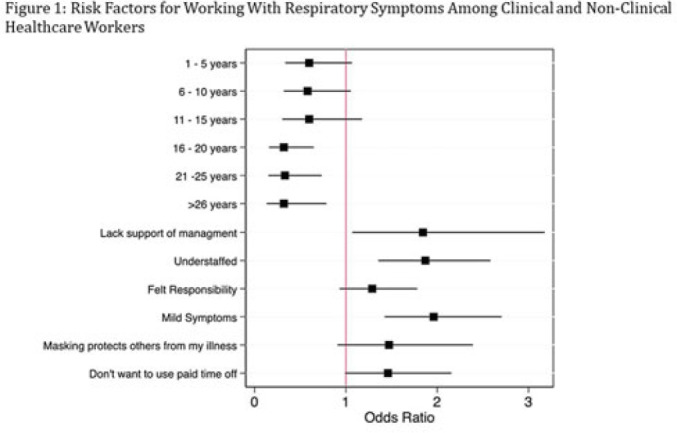

**Figure:**
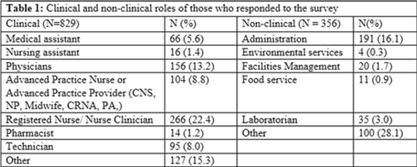

**Figure:**
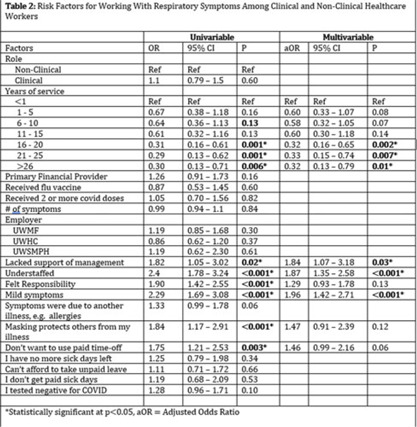

**Disclosures:** None

